# A ‘homemade’ snare for endovascular procedures

**DOI:** 10.1308/003588412X13373405385214l

**Published:** 2012-07

**Authors:** J Shalhoub, K Elliott, T Tran

**Affiliations:** North West London Hospitals NHS Trust,UK

## BACKGROUND

Snares have considerable utility during elective and urgent endovascular procedures.^1^ Endovascular snares are expensive, costing between £150–£200 per unit, and may not always be readily available when required, particularly in an urgent setting.

## TECHNIQUE

**Figure 1:**
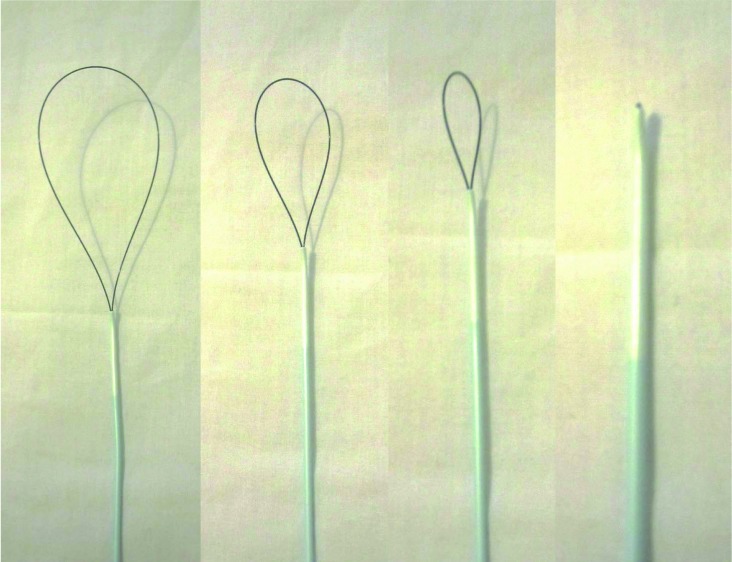
The ‘homemade’ endovascular snare, shown from open (left) to closed (right)

A ‘homemade’ snare can be fashioned using a 0.018” (0.46mm; external diameter) hydrophilic guidewire (eg ZIPwire®; Boston Scientific, Natick, MA, US; unit cost £18) and a 0.038” (0.97mm; internal diameter) endovascular catheter (eg Torcon NB®; Cook Medical, Bloomington, IN, US; unit cost £10). The stiff end of the guidewire is passed into the catheter until it emerges at the tip. It is then reversed and passed back into the tip to re-emerge at the catheter hub (Fig 1). The size of the snare is controlled by pulling on the two ends of the catheter at the hub.

## DISCUSSION

A 0.038” catheter accommodates a 0.038” guidewire snugly. The two ends of the 0.018” guidewire amount to 0.036”, allowing 0.002” (0.05mm) for ease of movement in the catheter. This approach may be used with a straight or curved-tipped catheter, the latter allowing the snare to be more easily ‘directed’ in the vessel. At a total cost of less than £30, this inexpensive snare is a helpful option during endovascular procedures.
